# Feedlot Factors Influencing the Incidence of Dark Cutting in Australian Grain-Fed Beef

**DOI:** 10.3390/ani12151989

**Published:** 2022-08-05

**Authors:** Cameron C. Steel, Angela M. Lees, Garth Tarr, Frank R. Dunshea, Des Bowler, Frances Cowley, Robyn D. Warner, Peter McGilchrist

**Affiliations:** 1 School of Environmental and Rural Science, University of New England, Armidale, NSW 2351, Australia; 2 Animal Science Group, School of Agriculture and Food Sciences, The University of Queensland, Gatton, QLD 4343, Australia; 3 School of Mathematics and Statistics, The University of Sydney, Sydney, NSW 2006, Australia; 4 Faculty of Veterinary and Agricultural Sciences, School of Agriculture and Food, The University of Melbourne, Melbourne, VIC 3010, Australia; 5 Faculty of Biological Sciences, The University of Leeds, Leeds LS2 9JT, UK; 6 Animal Health Data, 177 Bennetts Road, Norman Park, QLD 4170, Australia

**Keywords:** high pH, feedlot cattle, production factors, dark meat, weather

## Abstract

**Simple Summary:**

This study was conducted to investigate feedlot factors that influence the incidence of dark cutting in Australian grain-fed beef. Awareness of factors influencing dark cutting within the supply chain will enable the implementation of management strategies to manage dark cutting risk and reduce incidence. The reduction of dark cutting incidence will increase feedlot productivity as well as profitability across the entire supply chain.

**Abstract:**

It has been well-established that dark cutting (DC) is a multifactorial issue that is associated with numerous animal and management factors. However, there is limited understanding of the feedlot-based factors that contribute to the influence of DC. The aim of this study was to evaluate the effect of climate, animal, and feedlot factors on the incidence of pH non-compliance in Australian grain-fed cattle. For this study, feedlot and abattoir records from 142,228 individual cattle over a 1-year period were investigated. These data incorporated records from seven feedlots that consigned cattle to three abattoirs. The average incidence of DC in these carcasses was 2.8%. The production factors that were associated with increased risk of DC included feedlot, sex, hormone growth promotants (HGP), cattle health, and days on feed (DOF). Additionally, DC also increased by reduced solar radiation (SR, W/m^2^), lower wind speeds (WS, m/s), increased ambient temperature (T_A_, °C), higher rainfall, a higher average temperature–humidity index (THI), and increased duration of time above heat-load-index threshold of 86 (HLI ≥ 86) during the 7 days prior to feedlot departure. This study identified the feedlot factors that increase the risk of DC from a feedlot-management perspective.

## 1. Introduction

In Australia, dark cutting (DC) beef is defined as a carcass that has an ultimate pH ≥ 5.7 as described by Meat Standards Australia (MSA) protocols. Dark cutting beef is associated with reduced meat quality; variable tenderness, reduced water-holding capacity, and shortened shelf life [1,2]. The dark colour and appearance has a negative quality perception by consumers, thus, beef feedlots have a monetary reduction applied from abattoirs to compensate for the reduced product quality [3]. Whilst the incidence of DC in grain-fed beef is low (<3%), Australia’s total number of grain-fed carcasses for the year ending December 2018 was 2,988,292, with an average carcass weight of 330 kg [4]. Based on these production metrics and an average deduction of 59 c/kg [5] for a DC carcass, the cost of DC to the Australian feedlot industry is estimated to be between AUD 8.7 to 14.5 million per annum, depending on the incidence of DC during an annual cycle.

The cause of high pH at grading is predominantly attributed to low muscle glycogen reserves at slaughter. Glycogen availability at slaughter is associated with the volume of glycogenesis on-farm and glycogenolysis during the pre-slaughter period [6]. Thus, DC is a multifactorial issue, and is influenced by numerous pre-slaughter factors including nutrition, stress, and exercise [3,6,7]. Grain-fed cattle are typically on a higher plane of nutrition compared to grass-fed cattle and, thus, have greater glycogen stores and, therefore, generally lower incidence of dark cutting [8]. Further speculation is that grain-fed cattle are more acclimated to various stressors; including trucking, machinery, and regular contact with humans. However, DC remains an important factor influencing the economic viability of the feedlot industry. Data pertaining to the abattoir factors influencing the incidence of dark cutting have been reported previously [9]. The effect of feedlot factors, including infrastructure and management, the use of hormone growth promotants (HGP), the number of days on feed (DOF), health management, transport time, and climatic conditions in the week before exiting the feedlot, on the incidence of DC in feedlot cattle, are discussed herein.

## 2. Materials and Methods

Data were obtained from seven individual feedlots (Feedlot A, Feedlot B, Feedlot C, Feedlot D, Feedlot E, Feedlot F, and Feedlot G) that consigned cattle to three abattoirs over a one-year period extending from 1 September 2017 to 31 August 2018. All three abattoirs (Abattoir A, Abattoir B, and Abattoir C) were supplied cattle from a minimum of two of these seven feedlots. In addition, one feedlot supplied two abattoirs. Abattoir A had cattle consigned from Feedlot D and Feedlot E; Abattoir B received cattle from Feedlot A, Feedlot C, and Feedlot G; and Abattoir C processed cattle consigned from Feedlot B, Feedlot C, and Feedlot F (Table 1).

### 2.1. Feedlot Management Data

Feedlot data was captured through data management systems, per the respective management protocols for each individual feedlot (*n* = 7). These systems consisted of an integrated (*n* = 4) or ‘in-house’ (*n* = 3) data management system. Data from the feedlots consisted of:

- Lot no.: Number assigned to a group of cattle.
- NLIS: National livestock identification system number for each individual animal.
- Entry weight: First recorded weight of an individual animal on entry to feedlot at induction (kg).
- Exit weight: Last recorded weight of an individual animal before or on exit date (kg).
- Induction date: Date cattle entered feedlot.
- Exit date: Date cattle exited feedlot.
- Days on Feed (DOF): Exit date–induction date. Included as 10-day increments in models.
- Average daily gain (ADG): The average daily gain in kg for an animal over the feedlot period. Calculated by (exit weight–entry weight)/DOF: the total amount of days between induction date and exit date.
- Feedlot Morbidity; (Yes/No): If an animal was removed to the hospital pen for reasons such as injury, illness, or shy feeding it was counted as morbid.
- Exit time: Exit time from feedlot. Taken from the National Vendor Declaration forms sent to abattoir with each lot of cattle. These were then recorded into excel from either photographs or scanned copies of the NVDS. These were aligned with MSA feedback data using the variables kill date, feedlot, and number of head.
- Arrival time: Arrival time of cattle to the abattoir. Taken from photographs or scans of hand-written trucking sheets for groups of cattle entering the processing abattoir. These were aligned with the MSA feedback data using the variables kill date, feedlot, and number of head.
- Transport time = Arrival time at abattoir–exit time at feedlot.
- Time since last draft.
- Cattle washed before exit Yes/No.
- Comingling or combining lots before exit.
- Date of last draft.
- Truck Type; b-double or semi.

### 2.2. Meat Standards Australia Carcass Data

Carcass grading data from all feedlots was sourced directly from MSA database. Carcass data were obtained for the period encompassing 1 September 2017 to 31 August 2018. There were a total of 142,228 cattle consigned across the three abattoirs during the study period. Data exported from the MSA database included if carcasses had been treated with a hormone growth promotant (HGP, yes versus no) and sex, i.e., male versus female.

Carcasses were evaluated for MSA grading and AUS-MEAT chiller assessment guidelines by MSA-accredited graders (Meat Standards Australia 2007). The two specific carcass measurements utilised in this analysis were:

- Hump height, which is measured in 5 mm gradients and is primarily used to verify the tropical breed content declared on the vendor declaration (Meat Standards Australia 2007).
- Ultimate pH (pH_u_) and loin temperature, which is measured in the rib eye muscle (*longissimus thoracis)* of the chilled carcass at the quartering site approximately 12–48 h post-mortem. Temperature and pH were measured using an MSA-approved TPS MC-80 or TPS WP-80 M ph meter (TPS Pty Ltd., Brisbane, Australia). The pH was used to determine the incidence of DC, where carcasses with a pH ≥ 5.7 at the time of grading are classified as a DC.

### 2.3. Climatic Data

At each feedlot, climatic data were obtained at 15 min intervals via onsite weather stations (Davis Pro V2, Hayward, CA, USA) that were installed for the project. Weather data collected included solar radiation (SR; W/m^2^), wind speed (WS;m/s), rain (mm), relative humidity (RH), and ambient temperature (T_A_; °C). From these data, the temperature–humidity index (THI), heat-load index (HLI), and accumulated heat load (AHL) were calculated. The THI was calculated using the following equation, as adapted from Thom [10]:

$$\mathrm{THI}=0.8\times T_{A}\;\lfloor (\frac{\mathrm{RH}}{100}\times \left(T_{A}-14.4\right)\rfloor +46.4$$

where RH = relative humidity (%) and *T_A_* = wet-bulb or dew-point temperature.

The HLI was calculated using the equation described by Gaughan et al. [11], where the HLI equation takes the following forms:

(i) A nonlinear regression that applies when BGT is greater than 25 °C

HLI_BGT>25_ = 8.62 + (0.38 × RH) + (1.55 × BGT)−(0.5 × WS) + [e^2.4−WS^]

(ii) A linear model that applies when BGT falls below 25 °C

HLI_BGT<25_ = 10.66 + (0.28 × RH) + (1.3 × BGT)−WS

where RH = relative humidity (%); BGT = black globe temperature (°C); WS = wind speed (m/s); and e = the base of the natural logarithm (approximate value of e = 2.71828).

To calculate AHL, [11] established the following equations:

(i) if

[HLI_ACC_ < HLI_Lower Threshold_, (HLI_ACC_−HLI_Lower Threshold_)/M];

and

(ii) if

[HLI_ACC_ > HLI_Upper Threshold_, (HLI_ACC_−HLI_Upper Threshold_)/M, 0]

where HLI_ACC_ = the actual HLI value at a point in time; HLI_Lower Threshold_ = the HLI lower threshold where cattle will dissipate heat (e.g., 77); HLI_Upper Threshold_ = the HLI upper threshold where cattle will gain heat (e.g., 86); and M = number of measures per hour, i.e., number of times HLI data are collected per hour; if every 10 min, then M = 6 [11].

The HLI and AHL were evaluated on the threshold of 86, which describes the reference animal as defined by Gaughan et al. [11], specifically ra clinically healthy un-shaded black Angus steer < 100 days on feed.

### 2.4. Statistical Analysis

All data management and analysis were performed in R [12]. Data merging and manipulation were performed using the ‘dplyr’ package [13], whilst exploratory visualisations were generated within the ‘ggplot2’ package [14], and summary tables were generated using the package ‘table1’ [15]. In addition, calendar plots summarising climatic data were generated using the ‘sugrrants’ package [16]. Time series were conducted utilising the ‘tsibble’ package [16]. Where possible, data were merged at an individual animal level, via their unique national livestock identification system (NLIS) number.

To test the effect of production and management factors, generalised linear models with a logistic link function were fitted to indicator variable DC as the dependent variable, such that the estimated coefficients may be interpreted as log-odds or odds ratios when exponentiated. Abattoir and feedlot were included as fixed effects.

To explore the relationship between climatic variables and DC during the 7 days prior to feedlot departure and DC carcasses, three climatic models were fitted using generalised linear mixed models using the ‘lme4’ package [17]. Climatic data from Feedlot A, Feedlot E, and Feedlot F were deemed erroneous, and, as such, these feedlots were excluded from analyses investigating the influence of climatic variables on DC. The climatic effect models, sex, and HGP status were included as fixed effects, while feedlot and slaughter dates were included as random effects. **Model 1**, included SR, WS, rain, RH, and T_A_; **Model 2** incorporated SR, WS, rain, and THI; and **Model 3** included rain and HLI. Three models for the climatic data were needed due to the interdependence of climatic variables within the THI and HLI models. Specifically, the HLI is a function of black globe temperature, RH, and SR [12], thus, an inter-relationship between the climatic variables investigated exists. Furthermore, data evaluated within model 3 is not directly comparable to models 1 and 2, as there were fewer observations where HLI ≥ 86.

Model outputs were visualised and tabulated using the ‘sjPlot’ package [18]. This includes the forest plots for coefficients and tables of estimated odds ratios. Post hoc pairwise difference estimates were found using the ‘emmeans’ package [19].

## 3. Results

Feedlot D supplied the greatest number of cattle (44.3%), followed by Feedlot G (13.6%), Feedlot C (13.5%), and Feedlot A (13.2%) (Table 2). Average days on feed (DOF) across feedlots was 128 ± 76 days. Feedlot A had the highest mean DOF (285 ± 92 days, Table 3, whilst Feedlot F had the lowest mean DOF (61 ± 3 days, Table 3). Increasing DOF was associated with a slightly higher incidence of the carcass being classified as DC. A 10-day increase in DOF increased the odds of DC by 1.02 times (Table 4). In addition, feedlot morbidity was significant when included in the base model (*p* < 0.001) and was associated with an increased likelihood of DC. If an animal was identified to have received health treatment at the feedlot, they were 1.34 times more likely to have DC than their healthy (non-treated) peers.

### 3.1. Dark-Cutting Incidence by Feedlot

Feedlot G had the highest incidence of non-compliant MSA (5.61%), followed by Feedlot B (3.26%) and Feedlot D (3.12%; Table 3), whereas the lowest incidence of non-compliance was observed from Feedlot E (1.16%; Table 2). The influence of feedlot on DC was significant within the base model (Table 4). Cattle supplied by Feedlot D were 2.21 times more likely to be DC, when compared with cattle supplied from Feedlot A (*p* < 0.001). Conversely, cattle supplied from Feedlot F were 0.51 times less likely to be DC, when compared with the odds of Feedlot A (*p* = 0.001). The odds of carcasses being classified as DC from Feedlot C (*p* = 0.76), Feedlot B (*p* = 0.21), or Feedlot G (*p* = 0.86) were not different from Feedlot A.

### 3.2. Influence of Hormone Growth Promotants

A total of 72.7% of cattle within this study had been treated with HGP (Table 5). Feedlot B and Feedlot F had an HGP-usage rate of 100%, whereas Feedlot A did not use HGP (Table 5). Hence, for the analysis, these three feedlots and HGP status are confounded. The cattle implanted with HGP had DC incidence of 3.33%, whereas HGP-free cattle had an incidence of 1.68% (Table 6). Cattle implanted with HGP were 2.29 times more likely to be classed as DC, when compared with cattle that were not treated with HGP (*p* < 0.001; Table 4).

### 3.3. Influence of Sex

Males accounted for 71.5% of cattle within the study (Table 7). Feedlot B fed a higher proportion of female cattle (96.6%), whereas Feedlot G (92.7%) and Feedlot E (99.9%) fed a higher proportion of male cattle (Table 7). Female and male carcasses had a DC incidence of 3.21% and 2.86%, respectively. Although sex was significant in the model, the odds ratio for males being classified as DC was only slightly greater than the odds ratio for females, when evaluated in the base model (1.1458, *p* = 0.008; Table 2). When feedlot was removed from the baseline model, i.e., removing confounding interaction of feedlot and sex, males had a lower DC risk when compared with females.

### 3.4. Time in Transport

**Figure 1:**
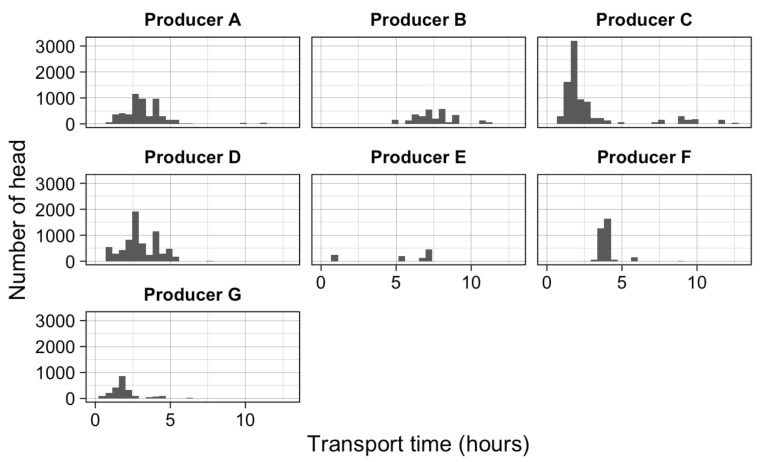
Transport duration (hours) for all feedlots.

Transport time was not associated with DC (*p* = 0.927). All of the transport times for Feedlot B (≈ 5 to 9 h), Feedlot D (≈ 1 to 5 h), Feedlot E (≈ 5 to 7 h), and Feedlot F (≈ 4 h) were similar for each lot of cattle (Figure 1). Feedlot C supplied Abattoir B and Abattoir C and, as such, had a greater variability in transport duration; however, the duration of transport was predominantly around 2 h (Figure 1).

### 3.5. Climatic Factors Influencing Dark Cutting

#### 3.5.1. Climate Model 1: Solar Radiation, Wind Speed, Rain, Relative Humidity, and Ambient Temperature

Solar radiation, WS, rain, and average T_A_ influenced the odds of carcasses being classified as DC (Table 8). There was no relationship between RH (*p* = 0.40) and T_A_ (range, *p* = 0.91; minimum T_A_, *p* = 0. 20; or maximum T_A_, *p* = 0. 15) and odds of DC within this model (Table 8).

Cattle exposed to a higher-average SR during the 7 days prior to feedlot departure had a slightly lower likelihood of being classified DC, when compared to animals that were exposed to lower SR (*p* < 0.05, Table 8). The odds ratio for DC for average SR was 0.997 times the odds of failing on pH, if exposed to 1 W/m^2^ less over the 7 days prior to feedlot departure. Similarly, higher average WS over the 7 days prior to feedlot departure were associated with lower odds of being classified DC (*p* < 0.05, Table 8). Cattle exposed to an average WS 1 m/s faster over the 7 days had 0.961 times the odds of DC, when compared with an animal with that had an average WS 1 m/s slower. Increased rainfall during the 7 days prior to feedlot departure increased the odds of DC (*p* < 0.001, Table 8), where cattle that experienced 10 mm of rain more had 1.13 times the odds of DC, when compared to cattle that were exposed to 10 mm less rainfall within the same period. Higher average T_A_ during the 7 days prior to feedlot departure correlated with an increase in the odds of pH non-compliance (*p* < 0.05, Table 8). Cattle that were exposed to an increased average T_A_ of 1 °C had 1.031 times the odds of DC, when compared with cattle exposed to average T_A_ that were 1 °C lower during the 7 days prior to feedlot departure.

#### 3.5.2. Climate Model 2: Solar Radiation, Wind Speed, Rain, and Temperature–Humidity Index

Solar radiation, WS, and rain influenced the odds of carcasses being classified as pH non-compliant (Table 9).

An increase in average THI during the 7 days prior to feedlot departure was associated with an increase in the odds of DC carcasses. A one-unit increase in THI was associated with 1.025 times the odds of DC, when compared to cattle that were exposed to conditions with a one-unit lower THI (*p* < 0.05). An increase in one unit of maximum THI increased the odds of DC by 1.026. There was no effect of increasing range, minimum THI, or maximum THI on DC (Table 9).

Similar to Climate Model 1, an elevation in SR during the 7 days prior to feedlot departure was associated with lowered odds of DC carcasses. The odds ratio for carcasses being classified as DC was 0.9967 times the odds of cattle exposed to SR 1 W/m^2^ more, during the 7 days before exit (*p* < 0.01). Faster average wind speeds during the 7-day period prior to feedlot departure were associated with lower odds of pH non-compliance. Cattle exposed to an average WS 1 m/s faster had 0.9621 times the odds of having a DC carcass, when compared to an animal that was exposed to an average WS of 1 m/s less (*p* < 0.05). An increase in rain was associated with an increase in odds of DC. Each 1 mm increase in rain was associated with a 1.013 increase in the odds of DC, compared with cattle exposed to 1 mm less rain during the 7 days prior to feedlot departure (*p* < 0.001).

#### 3.5.3. Climate Model 3: Rain and Heat-Load Index

An increase in the number of hours ≥ HLI_86_ during the 7 days prior to feedlot departure was associated with increased odds of DC (Table 10). Cattle that were exposed to 1 h longer per day of HLI ≥ 86 over the 7-day period had 1.0118 times the odds for DC, when compared to cattle that were exposed to HLI ≥ 86 for 1 h less during the 7 days prior to feedlot departure. Average (*p* = 0.50), maximum (*p* = 0.75), and minimum (*p* = 0.53) HLI were not associated with an increased likelihood of DC. Additionally, within this model, rain did not influence the likelihood of DC (*p* > 0.05).

## 4. Discussion

### 4.1. Feedlot Morbidity

Feedlot morbidity was associated with an increased incidence of DC in the current study. This was an anticipated outcome, as these cattle had been taken from their home pens to a health facility for assessment and treated for a health ailment. As such, these animals were exposed to a number of stressors including increased handling and exposure to external stimuli in a novel environment, so they become more stressed and utilise more glycogen [19]. If repeat treatments are required, cattle are relocated to a hospital pen, further increasing the duration of exposure to stressful stimuli. Hospital pens will usually hold cattle from different pens, hence, the cattle then establish social regrouping with each introduction of a new animal [20]. The stress involved with regrouping when mixing cattle has been shown to directly impact muscle glycogen utilisation [21]. Although not identified within this study, the underlying cause of why cattle were pulled to the hospital pen, such as sickness, injury, and disease, is likely to influence dry matter intake. It is well-established in the literature that decreases in feed intake or fasting will increase the incidence of pH non-compliance in grain-fed cattle, much like those on a lower plane of nutrition [22,23]. Within the current study, morbidities were defined as any animal that received treatment for a health ailment, and, as such, will include cattle that were treated and returned to their original home pens. Therefore, the higher odds of DC in these cattle may indicate that the impact of their underlying health issues may have persisted for the remainder of the feeding term.

### 4.2. Climatic Factors Influencing Dark Cutting

Increasing average T_A_, rain, THI, and hours where HLI ≥ 86 during the 7 days prior to feedlot departure were all associated with an increased incidence of DC. However, their impact relative to feedlot, abattoir, DOF, sex, and HGP were small. Conversely, increased average WS and increasing SR were associated with a decreased incidence of DC. This is not unexpected, as WS is well-known to influence thermal-exchange mechanisms [23] and increases the effectiveness of convective heat dissipation [24,25] (Berman 2005). Additionally, periods of heat load are well-documented to decrease in feed intake [26,27,28,29]. Furthermore, as heat load increases, cattle divert energy that is typically partitioned for growth towards maintaining homeostasis [28,30] Maintenance energy requirements are estimated to increase by 7% to 25% during hot climatic conditions. This diversion of energy towards homeostasis typically manifests as a reduction in feed conversion efficiency and reduced live weight gain [31]. During hot conditions, the reduction in feed intake, exposure to stressors, and redistribution of energy may result in lower muscle glycogen stores prior to feedlot exit, thus increasing the odds of DC. However, further studies are required to examine the relationship between carcass attributes and climatic conditions in cattle. Furthermore, the influence of climatic conditions and the intensity, time of exposure, and duration of exposure to both hot and cold conditions and the subsequent incidence of DC is yet to be established, requiring considerably more micro-climatic observations than what was assessed in this study.

### 4.3. Hormone Growth Promotants

The use of HGP within this study was associated with an increased risk of DC, which is in support of previous research [32,33,34,35]. Previous studies have established that the impact of HGP on DC is influenced by the type of HGP, the timing of its use, whether it was incorrectly implanted, and if over-dosing occurred. Aggressive use of HGP [32] or false-implant strategies can increase the susceptibility of cattle to stress and has been associated with a predisposition to DC, particularly when exposed to unusually stressful circumstances [34]. Although not well-established, there may also be an underlying influence of HGP on stress susceptibility in steers and heifers [36]. Steers treated with an androgen HGP may exhibit an increase in aggressive behaviour [35], which could increase the incidence of DC due to increased stress and muscle contraction. This effect will be amplified, if the steers are transported to slaughter while the HGP is still actively secreting hormone, or if it remains within the ‘pay-out period’ [35]. However, the impact of HGP on DC may be managed by slaughtering cattle 100 days after implantation [35]. Therefore, recommendations provided by HGP manufacturers should be strictly adhered to in order to minimise the influence that HGP may have on DC.

### 4.4. Sex

In the present analysis, steers had slightly higher odds of DC when compared with heifers. This result is partially supported by the findings of Page, et al. [37], who found no difference in muscle pH between steers and heifers. In the present study, the relationship was likely confounded by sex and feedlot, as once feedlot was removed from the base model, steers had a lower DC risk than heifers. The lower incidence of DC in steers is well-documented, as numerous studies have described steers to have lower incidences of DC when compared with females [33,38,39,40,41]. When heifers are exhibiting oestrus, glycogen-depleting activities such as increased mounting and walking are likely to account for the increased incidence of DC in female carcasses [40,42,43]. Furthermore, the sex effect on DC may also result from differences in stresses experienced by heifers, predisposition to stress, and hormonal fluctuations. Studies have found that there is a significant association between sex and temperament in cattle [44], and heifers tend to be more excitable and more fearful when compared with steers [38]. This highlights that females may be more susceptible to stress and, as such, are more likely to be classified as DC. Overall, this suggests that heifers need to be treated with greater care and all efforts should be made to minimise the pre-slaughter period and lairage time, reducing the opportunity for cycling animals to generate excitement, stress, and physical activity, which all impact glycogen depletion. When castrates are introduced into a group of females, increased physical activity resulting from mounting, chasing, and excitement increases muscle contraction and, therefore, depletes muscle glycogen stores at a higher rate than if castrates and females are maintained in separate pens. This excitement could be the reason why males in this base-model analysis had a higher odds ratio of DC.

### 4.5. Days on Feed

Increasing DOF was associated with a slight increase in the incidence of pH non-compliance in this study. This could be due to fibre types becoming more oxidative, i.e., slow twitch red type, in older animals; therefore, the term could be explaining further variation that ossification is not able to account for [45]. Days on feed has previously been shown to be associated with reduced insulin sensitivity [46]. As animals get older and fatter, their insulin sensitivity often declines, but, more importantly, their adrenaline sensitivity increases, suggesting that these animals utilise a greater proportion of glycogen during the pre-slaughter period, which has previously been demonstrated in sheep [47]. It is also possible that some of this variation could be explained by mixing pens of cattle. Cattle that are yet to reach market specifications, due to delayed growth, may remain at the feedlot. In these instances, cattle are typically reallocated to pens until market specifications are achieved [48]. It is possible that these cattle already have an increased likelihood of DC because of these stressors associated with regrouping, rather than an increased DOF.

### 4.6. Transport Time

While transport time did not increase the likelihood of DC, this can be partly explained by the limited variation in transport times across the seven feedlots in the current study. This is supported by the findings of Ferguson et al. [49], who found that transporting cattle < 400 km had no influence on the incidence of DC. Similarly, Chulayo, et al. [50] reported that cattle can acclimate during a 200–400 km commute but seem to subsequently have stress increases after this distance. The lack of effect of transport distance on DC within the current study could also be associated with these cattle being ≥ 100 DOF. It has been well-documented that grain-fed cattle have ample muscle glycogen prior to feedlot departure [7,19]. In addition, it is probable that feedlot cattle have previous experience with trucking, as they were transported from their home property to the feedlot.

## 5. Conclusions

The results from the current study suggest that there are numerous feedlot factors associated with the incidence of DC. Anticipated DC was variable across the seven feedlots, ranging between 1.16% and 3.26%. Numerous feedlot factors increased the odds of DC including health status, HGP status, sex, and climatic conditions. Feedlot climatic conditions had a significant effect on DC, although the overall effect was small. However, increasing heat load during the 7 days prior to feedlot departure was associated with small increases in DC, indicating that climatic conditions have an inherent role in DC. However, within the current study feedlot, abattoir, health status, HGP status, and sex were the predominant risk factors of DC, while transport time was not significant. Overall, these results provide further evidence that DC remains a multifactorial issue existing within specific supply chains, even for the Australian feedlot industry. Further investigation into feedlot health morbidity, confounded by disease or illness type within this study, would be beneficial to identify specific illnesses and their comparative influence on DC. Treating for specific underlying health issues could assist in reducing the incidence of DC throughout the feedlot supply chain.

## Figures and Tables

**Table 1:** Number of carcasses with attributes across seven feedlots and three processing facilities.

Feedlot	A (*n* = 7472)	B (*n* = 18,546)	C (*n* = 18,989)	D (*n* = 62,349)	E (*n* = 6082)	F (*n* = 8237)	G (*n* = 19,147)	Overall (*n* = 140,822)
Abattoir								
A	0 (0%)	0 (0%)	0 (0%)	62,349 (100%)	6082 (100%)	0 (0%)	0 (0%)	68,431 (48.6%)
B	0 (0%)	18,546 (100%)	4000 (21.1%)	0 (0%)	0 (0%)	0 (0%)	19,147 (100%)	41,693 (29.6%)
C	7472 (100%)	0 (0%)	14,989 (78.9%)	0 (0%)	0 (0%)	8237 (100%)	0 (0%)	30,698 (21.8%)
HGP								
Y	7472 (100%)	0 (0%)	15,899 (83.7%)	55,129 (88.4%)	5741 (94.4%)	8237 (100%)	9933 (51.9%)	102,411 (72.7%)
N	0 (0%)	18,546 (100%)	3090 (16.3%)	7220 (11.6%)	341 (5.6%)	0 (0%)	9214 (48.1%)	38,411 (27.3%)
SEX								
F	7221 (96.6%)	2488 (13.4%)	14,166 (74.6%)	10,105 (16.2%)	6 (0.1%)	4756 (57.7%)	1401 (7.3%)	40,143 (28.5%)
M	251 (3.4%)	16,058 (86.6%)	4823 (25.4%)	52,244 (83.8%)	6076 (99.9%)	3481 (42.3%)	17,746 (92.7%)	100,679 (71.5%)
DAYS ON FEED								
Mean (SD)	82.2 (17.0)	285 (92.0)	96.3 (36.3)	105 (25.3)	98.2 (7.07)	61.0 (3.05)	136 (38.2)	128 (76.3)
Median [Min, Max]	82.0 [8.00, 279]	223 [22.0, 565]	83.0 [43.0, 256]	103 [8.00, 282]	100 [70.0, 100]	60.0 [60.0, 70.0]	134 [69.0, 440]	103 [8.00, 565]

**Table 2:** The total number of carcasses, number of carcasses classified as compliant (pH ≤ 5.7) and non-compliant (pH > 5.70), and the percentage of non-complaint carcasses across the seven feedlots.

Feedlot	Total Carcasses	Compliant	DC	DC, %
A	18,546	18,314	232	1.27%
B	7472	7236	236	3.26%
C	18,989	18,510	479	2.59%
D	62,349	60,462	1887	3.12%
E	6082	6012	70	1.16%
F	8237	8102	135	1.67%
G	19,147	18,130	1017	5.61%

**Table 3:** The mean, median, minimum, and maximum days on feed for cattle from each feedlot.

Item	Feedlot						
	A	B	C	D	E	F	G
Mean	285 ± 92	82 ± 17	96 ± 36	105 ± 25	98 ± 7	61 ± 3	136 ± 38
Median	223	82	83	103	100	60	134
Minimum	22	8	43	8	70	60	69
Maximum	565	279	256	282	100	70	440

**Table 4:** Odds ratios of pH non-compliance for predictors including significant variables.

Predictors	Odds Ratio	Confidence Interval	Significance
Intercept	0.197	0.131–0.294	*p* < 0.001
DOF *	1.02	1.009–1.030	*p* < 0.001
HGP Yes	2.29	2.034–2.584	*p* < 0.001
Sex (steer)	1.14	1.036–1.267	*p* = 0.008
Abattoir 2 *	3.66	2.491–5.374	*p* < 0.001
Abattoir 3	0.88	0.572–1.353	*p* = 0.56
Feedlot B	1.27	0.876–1.829	*p* = 0.21
Feedlot C	0.95	0.682–1.322	*p* = 0.76
Feedlot D	2.21	1.729–2.816	*p* < 0.001
Feedlot F	0.51	0.345–0.759	*p* = 0.001
Feedlot G	0.97	0.744–1.276	*p* = 0.86

* DOF effect per 10-day increment. * Abattoir traits were included in the model and have been presented previously [8].

**Table 5:** Number of cattle implanted with hormone growth promotants (HGP; %) across the seven feedlots during the study.

HGP	Feedlot							Overall HGP
	A	B	C	D	E	F	G	
Yes	0 (0%)	7472 (100%)	15,899 (83.7%)	55,129 (88.4%)	5741 (94.4%)	8237 (100%)	9933 (51.9%)	72.7%
No	18,546 (100%)	0 (0%)	3090 (16.3%)	7220 (11.6%)	341 (5.6%)	0 (0%)	9214 (48.1%)	27.3%

**Table 6:** Number of carcasses classified as compliant (pH ≤ 5.69) and non-compliant (pH ≥ 5.70), according to hormone growth promotant usage.

HGP	Total Carcasses	Compliant	Non-Compliant	Proportion Non-Compliant
No	38,411	37,765	646	1.68%
Yes	102,411	99,001	3410	3.33%

**Table 7:** Distribution of female and male cattle across the seven feedlots during the study.

Sex	Feedlot							Overall
	A	B	C	D	E	F	G	
Female	2488 (13.4%)	7221 (96.6%)	14,166 (74.6%)	10,105 (16.2%)	6 (0.1%)	4756 (57.7%)	1401 (7.3%)	28.5%
Male	16,058 (86.6%)	251 (3.4%)	4823 (25.4%)	52,244 (83.8%)	6076 (99.9%)	3481 (42.3%)	17,746 (92.7%)	71.5%

**Table 8:** The odds ratios for the effect of the mean, range, max, and min for ambient temperature (T_A_, °C), solar radiation (SR, W/m^2^), wind speed (WS, m^2^), relative humidity (RH), and rain (mm) on the incidence of pH non-compliance.

Predictors	Mean Model		Range Model		Max Model		Min Model	
	Odds Ratio	Significance	Odds Ratio	Significance	Odds Ratio	Significance	Odds Ratio	Significance
Intercept	0.0118	*p* < 0.001	0.0394	*p* < 0.001	0.0429	*p* < 0.001	0.0152	*p* < 0.001
SR_MEAN_	0.9970	*p* = 0.013	0.9986	*p* = 0.096			0.9980	*p* = 0.061
SR_MAX_					0.9988	*p* = 0.004		
WS_MEAN_	0.9611	*p* = 0.022	0.9875	*p* = 0.50				
WS_MAX_					0.9857	*p* = 0.085		
WS_MIN_							0.6480	*p* = 0.036
RH_MEAN_	1.0039	*p* = 0.40						
RH_RANGE_			0.9886	*p* = 0.020				
RH_MAX_					0.9926	*p* = 0.17		
RH_MIN_							1.0084	*p* = 0.12
T_A, MEAN_	1.0315	*p* = 0.035						
T_A, RANGE_			0.9982	*p* = 0.91				
T_A, MAX_					1.0207	*p* = 0.15		
T_A, MIN_							1.0151	*p* = 0.20
Rain	1.0129	*p* < 0.001	1.0140	*p* < 0.001	1.0123	*p* < 0.001	1.0135	*p* < 0.001

**Table 9:** The odds ratios for the effect of THI (mean, max, range, and min) plus solar radiation (SR W/m^2^), wind speed (WS m^2^), relative humidity (RH g/m^3^), and rain (mm) on the incidence of pH non-compliance.

Predictors	Mean Model		Range Model		Max Model		Min Model	
	Odds Ratio	Significance	Odds Ratio	Significance	Odds Ratio	Significance	Odds Ratio	Significance
(Intercept)	0.0058	<0.001	0.0269	<0.001	0.0065	<0.001	0.0147	<0.001
sr_avg_7	0.9967	0.004	0.9982	0.044			0.9975	0.018
ws_avg_7	0.9640	0.030	0.9704	0.071				
rain_7	1.0132	<0.001	1.0130	<0.001	1.0116	<0.001	1.0135	<0.001
thi_avg_7	1.0253	0.018						
thi_range_7			0.9900	0.311				
sr_max_7					0.9986	0.001		
ws_max_7					0.9840	0.046		
thi_max_7					1.0264	0.043		
ws_min_7							0.6848	0.067
thi_min_7							1.0097	0.181

**Table 10:** The odds ratios for the effect of heat-load index (HLI) mean, max, min, HLI_<70_^1^, and HLI_86_^2^ _plus_ rain (mm) on the incidence of pH non-compliance.

Predictors	Mean Model		Max Model		HLI_86_ Model		HLI_<70_ Model	
	Odds Ratio	Significance	Odds Ratio	Significance	Odds Ratio	Significance	Odds Ratio	Significance
Intercept	0.0105	*p* < 0.001	0.0182	*p* < 0.001	0.0137	*p* < 0.001	0.0110	*p* < 0.001
HLI_MEAN_	1.0064	*p* = 0.50						
HLI_MAX_			0.9980	*p* = 0.75				
HLI_≤70_^1^							1.9360	*p* = 0.53
HLI_≥86_^2^					1.0118	*p* = 0.011		
Rain	1.0020	*p* = 0.61	1.0025	*p* = 0.52	1.0019	*p* = 0.62	1.0236	*p* = 0.27

^1^ number of days where HLI was ≤70 for ≥6 h during the 7 days prior to feedlot departure. ^2^ number of days where HLI was ≥86 during the 7 days prior to feedlot departure.

## Data Availability

The data presented in this study are available on request from the corresponding author.
